# A cross-sectional study of geographic differences in health risk factors among young Australian adults: The role of socioeconomic position

**DOI:** 10.1186/1471-2458-14-1278

**Published:** 2014-12-15

**Authors:** Kira AE Patterson, Verity Cleland, Alison Venn, Leigh Blizzard, Seana Gall

**Affiliations:** Menzies Research Institute Tasmania, Private Bag 23, Hobart, 7001 Tasmania Australia

**Keywords:** Australia, Rural health, Health behaviours, Adults, Cross-sectional, Socioeconomic factors

## Abstract

**Background:**

It remains unclear why living outside of an urban environment affects aspects of health, particularly whether these differences can be explained by other factors such as socioeconomic position (SEP). The aim of this study was to compare health risk factors between metropolitan and non-metropolitan young Australian adults and examine whether socioeconomic position (SEP) mediates any differences.

**Methods:**

Cross-sectional data came from an Australia-wide sample of 26–36 year-olds (n = 2567). Information on demographic characteristics, smoking, alcohol consumption, diet, physical activity (PA, mins/week) and mental health were collected by questionnaire, BMI from measured height and weight and daily steps using pedometers. Metropolitan versus non-metropolitan residence was classified from addresses. SEP included individual-level (education, occupation) and area-level measures. Prevalence ratios and ratio of means were calculated using log binomial, log multinomial and linear regression techniques.

**Results:**

Non-metropolitan residents were less likely to meet 2 or more dietary guidelines, reported less leisure-time PA and active commuting but more occupational and domestic PA than metropolitan residents. Non-metropolitan women were more likely to smoke and be obese. No differences in mental health were found. After adjusting for SEP, differences remained significant except for leisure-time PA (men and women) and smoking (women).

**Conclusions:**

Living outside metropolitan areas was associated with more risk factors in these young adults. Individual SEP and area-level disadvantage generally did not explain these differences, suggesting that a focus on geographic location as its own social determinant of health, beyond SEP, is warranted.

## Background

People living in regional, rural and remote areas generally have poorer health than their urban counterparts, reflected in higher levels of mortality and chronic disease [1, 2]. Modifiable risk factors for chronic diseases include poor dietary behaviours, smoking, excessive alcohol use, and physical inactivity [3]. Additionally, depression and anxiety have also been shown to be independent risk factors for chronic disease, particularly cardiovascular diseases [4, 5]. One explanation for differences in health across different geographical areas may be differences in these risk factors. Studies have shown that living in a rural area, compared to living in an urban area, is associated with higher levels of physical inactivity [6, 7], increased smoking and alcohol consumption [8–10], poorer dietary behaviours [11, 12] and higher reports of suicide, despite similar levels of mental health disorders in urban and rural areas [13, 14]. Whilst the majority of published literature investigating multiple health risk factors according to geographic location has been conducted in the United States (US) [6–8, 11] and certain parts of Europe [9, 12], little is known outside of these areas.

Australia presents a unique context to examine the associations between geographic location and health risk factors due to the wide distribution of the population across diverse geographic regions [15]. The few peer-reviewed Australian studies investigating urban–rural differences in health risk factors have focussed particularly on women [16, 17] and on physical activity [18], with little information available for men. Recent national data from the Australian Institute of Health and Welfare (AIHW) and the Australian Bureau of Statistics (ABS) shows that people living in non-metropolitan areas were more likely to be daily smokers, overweight or obese, be insufficiently active and drink alcohol at levels that place them at risk of harm over their lifetime compared to those living in metropolitan areas [19]. Government policies in Australia and elsewhere have a focus on improving health for those living outside metropolitan areas; however, these are based largely on descriptive analyses without adjustment for important potential confounders, so it is unclear what aspects of non-metropolitan areas should be targeted.

One potential target to reduce urban–rural disparities in health is socioeconomic position (SEP). There is considerable evidence of an inverse relationship between SEP and health risk factors. For example, socioeconomic disadvantage is associated with lower physical activity, poorer diet, higher smoking and alcohol consumption and poorer psychological wellbeing [20]. In addition, people living outside metropolitan areas are typically of lower socioeconomic status and have lower incomes, are less educated, and there are higher rates of unemployment than those in metropolitan areas [21]. Therefore, as SEP is closely related with geographic area of residence it is possible that it explains urban–rural differences in health risk factors, but this is less well understood.

Some studies have found that urban–rural variations in health disappear after controlling for variables related to SEP [22–24]. These have generally focussed on mortality or specific diseases (e.g. cancer), rather than health-related risk factors. As such, the populations in existing studies tend to be older with little known about associations in younger adults. Therefore, it is less well understood whether poorer health-related risk factors observed outside metropolitan areas are attributable to individual SEP factors. This has important implications for government policies, because if SEP explains most of the metropolitan and non-metropolitan differences in health then programs focussing on addressing socioeconomic disadvantage across all geographic areas would be more appropriate than programs specifically targeting non-metropolitan locations.

This study aimed to: 1) compare health risk factors between young Australian adults living in metropolitan (major cities) and non-metropolitan (regional/rural) areas and 2) explore whether SEP explained any differences seen. Based upon previous peer-reviewed literature and Australian national data, discussed above, we hypothesised that 1) health-related risk factors would be more prevalent in non-metropolitan areas compared to metropolitan areas and 2) adjusting for SEP would explain any differences seen.

## Methods

### Procedure and participants

This study used cross-sectional data from the Childhood Determinants of Adult Health (CDAH) study, a follow-up of participants from the 1985 Australian Schools Health and Fitness Survey (ASHFS) [25]. CDAH data were collected in 2004–2006 (aged 26–36 years). Of the 8498 participants involved in ASHFS, 5170 enrolled to participate in the CDAH study. Of the 5170 that enrolled, 2900 completed questionnaires and 2410 attended one of 34 clinics around Australia (not all participants attended a clinic). The analysis for this study includes participants who had data on area of residence, health risk factors, SEP factors and other covariates (n = 2567). The final number included in some of the analyses is less than this due to missing data for some of the outcome variables.

Using baseline (1985) characteristics, those with follow-up data were more often female (54% participants versus 45% non-participants), from regional/rural areas (41% participants versus 34% non-participants), from higher SES postcodes (25% participants versus 22% non-participants) and were less likely to be smokers (12% participants versus 15% non-participants) in 1985 than those without follow up data. In the restricted sample of participants (n = 2567), those who had complete follow-up data were more often female (55% versus 52%), university educated (43% versus 29%), living in major cities (73% versus 66%) and never smokers (55% versus 47%) than those who did not have complete follow up data.

Compared with the general population of 25–34 year old Australians, a higher percentage of CDAH participants were married/living as married (71% versus 61%), were employed as professionals/managers (52% versus 39%) and were university-educated (40% versus 22%) [26] and a lower percentage were current smokers (22% versus 30%) [27]. The percentage classified as being overweight or obese (Body Mass Index ≥ 25) was very similar to the general population of the same age (48% versus 47%) [27].

### Measures

#### Area of residence classification

The Accessibility/Remoteness Index of Australia (ARIA+) is a well-established classification that is recognised as a nationally consistent measure of geographic remoteness and was used to define area of residence. It uses a standardised approach to classify ‘remoteness’ based on road distance measurements to services centres (more remote localities have lower access to service facilities). The Australian Bureau of Statistics (ABS) uses ARIA+ scores to classify remoteness areas in Australia as major cities, inner regional, outer regional, remote and very remote [28]. ARIA+ scores were assigned based on the ‘census collection district’ (CCD) of participant’s residential addresses. A CCD is one of the smallest spatial units available for data from the ABS, typically containing around 250 households. Due to small participant numbers in some of the ARIA+ categories, major cities were classified as metropolitan while inner regional, outer regional, remote and very remote areas were classified as non-metropolitan. The percentage of CDAH participants living in metropolitan and non-metropolitan areas was very similar to the general population of the same age (71% versus 74%; 29% versus 26%, respectively) [26].

#### Smoking status

Smoking status was self-reported via questionnaire, with participants classified as never smoker, ex-smoker, or current regular smoker [29].

#### Alcohol consumption

Self-reported alcohol consumption was measured using the food frequency questionnaire (FFQ). The FFQ asked about the average number of times each alcoholic beverage was consumed over the previous 12 months (from 10 common types of beverages). For each item (10 in total), participants were asked to choose one of nine responses ranging from “never or less than once a month” to “six or more times per day”. Daily alcohol consumption in grams was estimated from the usual frequency of consumption of the 10 common types of beverages over the previous 12 months multiplied by the average alcohol concentration of each beverage. Participants were categorised using recommended guidelines on alcohol consumption [30] as: none, 20 grams/day or less, or >20 grams/day.

#### Body mass index (BMI)

BMI (kg/m^2^) was calculated using clinically measured height and weight and categorised according to standard definitions of normal weight (<25 kg/m^2^), overweight (25–29.9 kg/m^2^) and obese (BMI ≥ 30 kg/m^2^) [31].

#### Self-reported physical activity

Physical activity was measured using the reliable and reasonably valid long version of the International Physical Activity Questionnaire (IPAQ-L) [32]. Participants self-reported duration (mins) and frequency (times/week) of occupational, domestic, commuting and leisure-time physical activity (LTPA). Minutes/week spent in each domain was calculated by multiplying frequency by duration. All reported physical activity was summed to provide an estimate of total minutes of past week physical activity.

#### Steps

Participants wore a Yamax Digiwalker pedometer (SW-200) and recorded total steps at the end of the day, daily start time and daily end time for seven consecutive days. Exclusion criteria and data management have been described elsewhere [31]. Within the sample for the current study (n = 2567), the overall response rate of those with pedometer data was 77% (n = 1971). The response rate of those with pedometer data from metropolitan areas and non-metropolitan areas was 78% and 77%, respectively.

#### Diet

Diet was assessed using a 127 item food-frequency questionnaire (FFQ). Participants reported how many times in the previous 12 months they consumed each item using a 9-point scale ranging from ‘never/less than once per month’ to ‘6 or more times per day’. The FFQ was a modified version of that used in the 1995 Australian National Nutrition Survey [33] and was based on an existing validated FFQ developed for Australian populations [34, 35]. Daily equivalents were calculated for each FFQ item and based on this information six dietary guideline variables were created, as described elsewhere [36]. The six guideline variables reflect the five core food groups (fruit, vegetables, dairy, breads and cereals, lean meats) and “extra” foods (those not included in the core food groups that are high in fat, salt and sugar).

#### Depression and anxiety

Depression and anxiety were measured using the validated Computerised International Diagnostic Interview (CIDI) [37], which was self-administered using a laptop computer at the study clinics.

#### Socioeconomic position

Education, occupation and employment status were used to measure individual SEP [38]. Participants self-reported their own highest level of education, their employment status and occupation. Education was collapsed into three categories: university (degree or higher); diploma/vocational/year 12 (certificate/diploma, trade/apprenticeship or year 12 or equivalent); and less than year 12 (all schooling up to the completion of Year 11). Occupation was collapsed into four categories: managers and professionals (managers and administrators, professionals and associate professionals); white collar (clerical, sales and service occupations); blue collar (trades, production and labourer positions); and not in labour force (retired, home duties, unemployed and students). Employment was collapsed into three categories: employed full-time; employed part-time; or other (student, home duties, retired or unemployed).

To measure area-level SEP, the ABS Index of Relative Socio-economic Disadvantage (IRSD) from the Socio-Economic Indexes for Areas (SEIFA) was used [39]. The IRSD uses census data to reflect the overall level of socioeconomic disadvantage of an area measured on the basis of attributes such as low educational attainment, low income, high unemployment, jobs in relatively unskilled occupations and high levels of public housing. A low score on this index indicates a high proportion of relatively disadvantaged people in an area. SEIFA scores were assigned at the level of CCDs based on participant’s residential address.

#### Other covariates

Other covariates included self-reported age, marital status (single, married/living as married, separated/divorced), parity for women and medical history. Self-reported medical history included information on hypertension, angina, heart attacks, stroke, high cholesterol, high triglycerides and diabetes. Participants were asked ‘Have you ever been told that you have’ any of the above conditions in which they could respond ‘yes’ or ‘no’ to.

### Analysis

Means with standard deviations and proportions were used to describe the socio-demographic characteristics and health risk factors of the sample, stratified by area of residence and sex. Comparisons between area of residence for men and women separately were performed using t-tests for continuous variables and chi-squared tests for categorical variables.

Associations between area of residence (study factor) and each health risk factor (outcome factor) were examined using log binomial regression (for variables with two categories), log multinomial regression (for variables with three or more categories) [40] and linear regression (for continuous variables). For categorical variables, prevalence ratios (PR) and 95% confidence intervals (CI) are reported. A PR of 1.10, for example, indicates that the prevalence in that group is 10% higher than the prevalence in the reference group. For continuous variables, ratios of means (ROM) and 95% CIs are reported. A ROM of 1.10, for example, indicates that the mean of that group is 10% higher than the mean of the reference group. Where necessary, continuous variables with skewed distributions were transformed (by taking logarithms) prior to analysis. For occupational physical activity by women, for which there was a large number of zero values (n = 762), a binary variable was created to reflect the proportions of active persons and those with no occupational activity. Log binomial regression was used to investigate differences between the ‘active’ and ‘not active’ groups and further linear regression analyses were restricted to the active group. The regression estimates are adjusted for age (model 1), additionally adjusted for individual SEP factors (model 2: one or more of education, occupation, employment status, marital status and parity in women), and additionally adjusted for area-level disadvantage (model 3). Adjustments for individual SEP factors and other covariates was made only if including a covariate for that outcome factor changed the estimated coefficient of area of residence by more than 10%. All models were checked for effect modification by all factors by including product terms as additional covariates. Results are shown separately for men and women because tests of interaction revealed significant differences. Analyses were conducted using STATA software (version 12.1, Statacorp, College Station, TX).

This study was a follow-up of individuals widely dispersed throughout many geographic locations in Australia rather than a study of selected neighbourhoods. Whilst a wide range of individual-level characteristics were measured, comprehensive information on neighbourhood characteristics was not gathered. The omission of neighbourhood-level covariates in a multi-level model would have caused the contribution of individual-level covariates to be overstated [41]. Instead of a multi-level analysis, we focused on a single-level analysis of individuals for which we had a rich collection of data.

### Ethical approval

Ethics approval was granted in 2004–6 by the Southern Tasmanian Health and Medical Human Research Ethics Committee and participants provided written informed consent.

## Results

### Sample

For both men and women, there were significant differences between metropolitan and non-metropolitan areas in education, marital status, occupation and SEIFA disadvantage (Table 1). For women, there were also significant differences in employment status and number of children.

**Table 1:** **Socio-demographic characteristics of men and women aged 26–36 years, by area of residence**

	Men (n = 1148)		Women (n = 1419)	
	Metropolitan	Non-metropolitan	Metropolitan	Non-metropolitan
Age (years), M (SD)	31.6 (2.6)	31.9 (2.5)	31.4 (2.6)	31.8 (2.5)
Education, % (n)				
University	42.7 (361)	28.1 (85)	50.9 (525)	37.0 (143)
Dip/voc/year12	48.1 (406)	58.7 (178)	41.0 (423)	43.9 (170)
<Year 12	9.2 (78)	13.2 (40)	8.1 (84)	19.1 (74)
	*p* = 0.001		*p* = 0.001	
Marital status, % (n)				
Single	31.7 (268)	23.4 (71)	26.4 (273)	14.5 (56)
Married/living as married	65.8 (556)	74.3 (225)	69.6 (718)	81.6 (316)
Separated/divorced	2.5 (21)	2.3 (7)	4.0 (41)	3.9 (15)
	*p* = 0.02		*p* = 0.001	
Occupation, % (n)				
Managers/professionals	62.8 (531)	48.5 (147)	54.2 (559)	40.6 (157)
White collar	7.9 (67)	6.3 (19)	25.2 (260)	27.4 (106)
Blue collar	25.7 (217)	42.6 (129)	4.2 (43)	6.7 (26)
Not in labour force	3.6 (30)	2.6 (8)	16.4 (170)	25.3 (98)
	*p* = 0.001		*p* = 0.001	
Employment status, % (n)				
Full-time	89.7 (758)	91.4 (277)	54.4 (561)	36.9 (143)
Part-time	5.4 (46)	5.3 (16)	24.9 (257)	34.9 (135)
Other	5.9 (41)	3.3 (10)	20.7 (214)	28.2 (109)
	*p* = 0.52		*p* = 0.001	
SEIFA disadvantage, M (SD)	1041.1 (70.1)	1002.2 (75.5)	1042.6 (70.4)	995.0 (74.6)
	*p* = 0.001		*p* = 0.001	
Number of children, % (n)				
None	-	-	55.3 (571)	26.6 (103)
One	-	-	18.5 (191)	19.4 (75)
Two	-	-	19.3 (199)	38.5 (149)
≥Three	-	-	6.9 (71)	15.5 (60)
			*p* = 0.001	

SD: standard deviation; DIP: diploma; VOC: vocational education; SEIFA: socioeconomic indexes for areas.

### Differences in health risk factors by area of residence

#### Men

Differences in risk factors were found between metropolitan and non-metropolitan men for physical activity and diet, but not for smoking, alcohol consumption, BMI, or anxiety and depression (Table 2). Men living in non-metropolitan areas reported significantly more occupational and domestic physical activity and more steps per day but reported significantly less active commuting and LTPA than men living in metropolitan areas. All associations (except LTPA) remained statistically significant when individual SEP factors and area-level disadvantage were taken into account. Men living in non-metropolitan areas on average reported 19% (95% CI: 6%, 31%) more minutes/week of total physical activity but, after adjustment for individual and area-level SEP factors, this association was reduced to 8% (95% CI: -4%, 19%) and was no longer significant. Men living in non-metropolitan areas were less likely to meet 2 or more dietary guidelines, even after adjusting for individual SEP and area-level disadvantage. Of the dietary behaviours examined, the only significant difference was for extra foods, where those in non-metropolitan areas consumed more serves per day of extra foods (β = 0.60 95% CI: 0.20, 1.00) than those in metropolitan areas. While non-metropolitan men consumed more bread, vegetables and dairy foods and less fruit and lean meats, these results were not statistically significant.

**Table 2:** Adjusted ratios (95% CI) of outcome risk factor* variables by area of residence for men

	Metropolitan ^a^	Non-metropolitan			
			Model adjusted for age	Model adjusted for age and individual SEP factors	Model adjusted for age, individual SEP factors and area-level disadvantage
	Mean (n or SD)	Mean (n or SD)	Ratio (95% CI)	Ratio (95% CI)	Ratio (95% CI)
Smoking status (n = 1144)					
Never	57.3% (484)	56.0% (168)			
Ex-smoker	17.6% (149)	20.0% (60)	1.12 (0.86, 1.47)	1.09 (0.83, 1.43)^c^	1.08 (0.82, 1.42)
Current smoker	25.0% (211)	24.0% (72)	0.96 (0.76, 1.21)	0.84 (0.67, 1.05)^c^	0.86 (0.69, 1.08)
Alcohol consumption (n = 1135)					
None	8.6% (72)	8.7% (26)			
20gm/day or less	75.8% (633)	72.7% (218)	0.96 (0.89, 1.04)	0.96 (0.89, 1.04)^d^	0.97 (0.89, 1.05)
More than 20gm/day	15.6% (130)	18.6% (56)	1.20 (0.90, 1.59)	1.20 (0.91, 1.58)^d^	1.25 (0.95, 1.65)
BMI (n = 1135)					
Not overweight	41.0% (345)	34.7% (102)			
Overweight	44.2% (372)	47.3% (139)	1.05 (0.91, 1.21)	1.02 (0.89, 1.18)^e^	1.03 (0.89, 1.19)
Obese	14.8% (124)	18.0% (53)	1.21 (0.90, 1.62)	1.08 (0.81, 1.44)^e^	1.05 (0.79, 1.40)
Physical activity (mins/week) (n = 1044)					
Occupational^b^	84.1 (227.1)	208.2 (395.3)	**2.45 (1.70, 3.21)**	**1.83 (1.29, 2.37)** ^**d**^	**1.74 (1.21, 2.28)**
Domestic^b^	92.9 (141.9)	132.8 (174.1)	**1.40 (1.13, 1.67)**	**1.34 (1.08, 1.60)** ^**d**^	**1.31 (1.05, 1.57)**
Active Commuting^b^	21.6 (64.5)	11.9 (41.4)	**0.55 (0.29, 0.81)**	**0.63 (0.34, 0.92)** ^**d**^	**0.61 (0.32, 0.90)**
Leisure time^b^	91.1 (163.5)	65.7 (131.8)	**0.73 (0.53, 0.93)**	0.84 (0.62, 1.06)^d^	0.88 (0.64, 1.11)
Steps per day (n = 903)^b^	8519.8 (3374.3)	9378.9 (3490.0)	**1.10 (1.04, 1.16)**	**1.07 (1.01, 1.13)** ^**d**^	**1.07 (1.01, 1.13)**
Dietary guideline met (n = 1096)					
Less than 2 guidelines	42.4% (341)	49.5% (144)			
2 or more guidelines (up to 5)	57.6% (464)	50.5% (147)	**0.87 (0.76, 0.99)**	**0.88 (0.78, 0.99)** ^**f**^	**0.88 (0.77, 0.99)**
Depression (n = 929)					
Negative	94.6% (695)	93.8% (182)			
Positive	5.4% (40)	6.2% (12)	1.13 (0.61, 2.12)	1.11 (0.59, 2.07)^c^	1.13 (0.59, 2.14)
Anxiety (n = 929)					
Negative	93.5% (687)	92.8% (180)			
Positive	6.5% (48)	7.2% (14)	1.10 (0.62, 1.96)	1.22 (0.68, 2.18)^e^	1.16 (0.63, 2.10)

CI: confidence interval; ref: referent; BMI: body mass index.

All bolded values are statistically significant at the 0.05 level.

*Sample sizes vary due to missing data for outcome variables (range 1,144 to 903).

^a^Metropolitan is the reference category.

^b^Data is summarised as mean (SD) and as ratios of means (95% CI).

^c^Adjusted for own highest level of education, occupation, marital status.

^d^Adjusted for own highest level of education, occupation.

^e^Adjusted for own highest level of education, occupation, marital status, employment status.

^f^Adjusted for own highest level of education.

#### Women

Women living in non-metropolitan areas were significantly less likely to be ex-smokers and to meet 2 or more dietary guidelines, but more likely to be current smokers and obese, than women living in metropolitan areas (Table 3). These associations remained statistically significant after adjusting for individual SEP. Further adjusting the models for area-level disadvantage did not explain differences seen for diet, obesity and being an ex-smoker, but the difference in proportions of current smokers was no longer statistically significant.

**Table 3:** **Adjusted ratios (95**% CI) of outcome risk factor* variables by area of residence for women

	Metropolitan ^a^	Non-metropolitan			
			Model adjusted for age	Model adjusted for age and individual SEP factors	Model adjusted for age, individual SEP factors and area-level disadvantage
	Mean (n or SD)	Mean (n or SD)	Ratio (95% CI)	Ratio (95% CI)	Ratio (95% CI)
Smoking status (n = 1418)					
Never	54.1% (558)	56.6% (219)			
Ex-smoker	26.0% (268)	18.9% (73)	**0.71 (0.56, 0.89)**	**0.63 (0.50, 0.80)** ^**c**^	**0.62 (0.49, 0.79)**
Current smoker	19.9% (205)	24.5% (95)	**1.25 (1.01, 1.54)**	**1.23 (1.00, 1.52)** ^**c**^	1.14 (0.92, 1.40)
Alcohol consumption (n = 1400)					
None	18.0% (183)	24.4% (94)			
20 gm/day or less	75.5% (766)	70.7% (272)	0.94 (0.87, 1.01)	1.02 (0.95, 1.10)^c^	1.04 (0.97, 1.13)
More than 20 gm/day	6.5% (66)	4.9% (19)	0.75 (0.45, 1.23)	0.90 (0.53, 1.54)^c^	0.95 (0.55, 1.64)
BMI (n = 1387)					
Not overweight	65.5% (670)	53.3% (194)			
Overweight	23.1% (236)	26.4% (96)	1.12 (0.91, 1.38)	1.03 (0.83, 1.28)^d^	1.07 (0.85, 1.34)
Obese	11.4% (117)	20.3% (74)	**1.75 (1.33, 2.28)**	**1.59 (1.20, 2.11)** ^**d**^	**1.46 (1.08, 1.96)**
Physical Activity (mins/week) (n = 1349)					
Occupational					
No activity	58.4% (573)	51.5% (189)			
Some activity	41.6% (409)	48.5% (178)	**1.18 (1.04, 1.34)**	**1.26 (1.11, 1.43)** ^**c**^	**1.23 (1.07, 1.40)**
Of those with some activity (n = 587)^b^	261.9 (303.7)	228.4 (265.5)	0.86 (0.68, 1.04)	0.88 (0.69, 1.06)^c^	0.82 (0.64, 1.00)
Domestic^b^	187.1 (246.6)	311.2 (314.4)	**1.61 (1.39, 1.82)**	**1.22 (1.06, 1.38)** ^**c**^	**1.16 (1.00, 1.33)**
Active Commuting^b^	44.7 (96.5)	24.2 (62.1)	**0.56 (0.39, 0.73)**	**0.62 (0.43, 0.81)** ^**c**^	**0.62 (0.42, 0.81)**
Leisure time^b^	96.0 (155.0)	63.2 (114.0)	**0.67 (0.52, 0.81)**	**0.79 (0.62, 0.97)** ^**c**^	0.84 (0.66, 1.03)
Steps per day (n = 1068)^b^	8543.7 (2975.8)	8506.4 (2996.8)	0.99 (0.94, 1.04)	0.99 (0.95, 1.04)^c^	1.00 (0.95, 1.05)
Dietary guideline met (n = 1344)					
Less than 2 guidelines	28.2% (275)	38.1% (141)			
2 or more guidelines (up to 5)	71.8% (699)	61.9% (229)	**0.86 (0.79, 0.94)**	**0.88 (0.81, 0.96)** ^**e**^	**0.91 (0.83, 0.99)**
Depression (n = 1056)					
Negative	89.4% (739)	86.9% (199)			
Positive	10.6% (88)	13.1% (30)	1.23 (0.83, 1.81)	1.14 (0.76, 1.71)^c^	1.06 (0.70, 1.61)
Anxiety (n = 1056)					
Negative	87.2% (721)	86.9% (199)			
Positive	12.8% (106)	13.1% (30)	1.02 (0.70, 1.49)	1.01 (0.68, 1.49)^c^	0.98 (0.65, 1.48)

CI: confidence interval; ref: referent; BMI: body mass index.

All bolded values are statistically significant at the 0.05 level.

*Sample sizes vary due to missing data for outcome variables (range 1,418 to 1,056).

^a^Metropolitan is the reference category.

^b^Data is summarised as mean (SD) and as ratios of means (95% CI).

^c^Adjusted for own highest level of education, occupation, marital status, employment status, number of children.

^d^Adjusted for own highest level of education, occupation, employment status, number of children.

^e^Adjusted for own highest level of education, occupation.

Women living in non-metropolitan areas were significantly more likely to be undertaking some occupational activity, and reported more domestic physical activity but less active commuting and LTPA, than women in metropolitan areas. The associations for occupational and domestic physical activity and active commuting remained statistically significant after adjustment for individual SEP and area-level disadvantage. The association for LTPA remained after adjusting for individual SEP but was no longer significant after adjustment for area-level disadvantage. There were no significant differences for total physical activity before and after adjustment for SEP factors (ROM: 1.07; 95% CI: 0.98, 1.16 and ROM: 0.97; 95% CI: 0.88, 1.06, respectively). There were also no significant differences for steps per day, alcohol consumption, or anxiety and depression. As with men, the only difference in dietary behaviours was for extra foods, where women living in non-metropolitan areas consumed significantly more serves per day of extra foods (β = 0.31 95% CI: 0.02, 0.60) than metropolitan women. Non-metropolitan women consumed more vegetables but less fruit, bread, dairy foods and lean meats, but differences were not statistically significant.

## Discussion

This study aimed to examine the differences in multiple health risk factors between residents of metropolitan and non-metropolitan areas and determine the role of SEP in any differences seen among young Australian adults. Our hypothesis regarding metropolitan-non-metropolitan patterning of health risk factors among young adults was largely supported. There was little support for our second hypothesis, with SEP generally not explaining the geographic differences in risk factors.

Non-metropolitan participants reported significantly more occupational and domestic physical activity but reported less active commuting and LTPA than people living in metropolitan areas. Previous studies investigating physical activity according to area of residence have generally focussed on LTPA or active commuting and have found urban adults report more LTPA and active commuting than rural adults [6, 7, 42]. We add to this literature by showing that those living outside metropolitan areas acquire more physical activity in other domains such as occupational and domestic physical activity than those living in metropolitan areas. Although those living in non-metropolitan areas report less LTPA and active commuting, greater activity in other domains for those living in non-metropolitan areas means both groups report similar amounts of total physical activity. This shows the importance of capturing and promoting physical activity within different domains.

Non-metropolitan participants were also less likely to meet 2 or more dietary guidelines and consume more serves per day of extra foods. This finding is supported by previous literature where people living in regional and rural areas have poorer dietary behaviours compared to those living in major cities [11, 12]. The higher cost of healthier foods [43], the availability of energy-dense nutrient-poor foods [44] and the decline in availability of basic healthy food items outside metropolitan areas and as remoteness increases in Australia [43] may lead to less healthful diets in non-metropolitan areas. Additional barriers such as lower levels of nutritional knowledge and lack of meal planning and food preparation skills may also lead to less healthful diets outside metropolitan areas [43].

Women living in non-metropolitan areas were more likely to be current smokers and obese than metropolitan women, independent of individual SEP. Again these findings are consistent with previous literature [8]. One study of women from Victoria, Australia, found that overweight and obesity were more common in rural than urban women; in contrast to the current study however, the differences were mostly explained by individual level socio-demographic characteristics [17]. Further, a study of US adults reported significantly higher prevalence of obesity in rural than urban adults, but the effect of rural residence remained significant after controlling for demographic composition [11].

This study found no significant differences in depression and anxiety between metropolitan and non-metropolitan men and women. These findings are consistent with other Australian–based and international studies [14, 22], which also found few differences in the prevalence of mental health disorders among metropolitan-non-metropolitan residents.

Controlling for both individual and area-level SEP did not eliminate the associations for dietary guidelines met, occupational and domestic physical activity, active commuting and steps per day for men and for women it did not explain the associations for active commuting, domestic and occupational physical activity, dietary guidelines met and being an ex-smoker and obese. This indicates that non-metropolitan residence is associated with these health risk factors above and beyond the effects of age, education level, occupation, employment status and marital status when compared to metropolitan residence. The differences that we observed in non-metropolitan areas could be due to unmeasured individual characteristics including other measures of SEP, the social or cultural environment or other complex spatial, economic or political factors which all warrant further investigation. Furthermore the built or physical environments related to non-metropolitan areas may also explain these patterns. This may include less access to preventative health services and staff [1], less availability of fresh fruit and vegetables and basic healthy food items [43], and less active commuting may be related to less infrastructure for walking, longer commuting routes and decreased access to public transportation in non-metropolitan areas [18]. In contrast, doing more occupational and domestic physical activity in non-metropolitan areas could be due to larger properties, yards and greater opportunity for physically demanding occupations but there is limited literature examining these domains of physical activity to support this. Whilst we are unable to disentangle the specific factors that contribute to these differences in the current study, our results suggest that geographic location is an important component of the social determinants of health.

Although the effects were modest, SEP did attenuate some associations. Adjustment for individual SEP eliminated the significant associations for LTPA and total physical activity for men. For women, the significant associations for LTPA and being a current smoker remained after adjustment for individual SEP and were only attenuated after further adjustment for area-level disadvantage. Given that smoking is a behaviour strongly patterned by SEP [45, 46] it is not surprising that the association for women was attenuated after adjustment for area-level disadvantage. While smoking is an individual behaviour, previous literature has shown that it is shaped by social context and is strongly related to social norms, in addition to individual socioeconomic factors [46]. Similarly, for LTPA, adults of lower SEP are commonly found to be less active in their leisure-time than adults of higher SEP [47]. Hence, this may explain why the associations between area of residence and LTPA in the current study disappear after taking SEP into account.

Limitations of this study include the cross-sectional analysis of the data, which excludes any conclusions regarding causality. The use of self-report measures may contribute to inaccuracy in the assessment of health risk factors; however, all measures used are widely accepted. Due to small participant numbers in some of the ARIA+ categories we had to categorise regional and rural areas as non-metropolitan areas, limiting the ability to look at regional and rural areas separately. However, the ABS has also used these same classifications (metropolitan versus non-metropolitan) to examine differences in health outside major cities [48] and the percentage of those living in metropolitan areas and non-metropolitans areas in the current study is similar to that of the general population. Although it was a national study, the sample was not strictly representative of the general population; therefore this may limit the generalisability of the prevalence estimates. Furthermore, given that this data was collected in 2004–06, it may not entirely reflect contemporary metropolitan-non-metropolitan differences in health risk factors. Lastly, information on whether the participants are of Aboriginal/Torres Strait Islander origin was not collected. Given the small proportion of people in the Australian population that identify as being of Aboriginal or Torres Strait Islander origin (2.5%) [49], it is unlikely to be an explanation for the differences observed.

There are also several strengths of the study. We had a large, national sample that included both men and women. We were able to examine a comprehensive range of health risk factors according to area of residence using well-established instruments, and were able to consider a large range of potential confounding factors in analyses. We were also able to examine the influence of both individual- and area-level SEP on health risk factors.

## Conclusion

This study identified differences in health risk factors between metropolitan and non-metropolitan areas, but these were not uniform across all of the health risk factors examined. Adults living in non-metropolitan areas demonstrated poorer health risk factors than adults living in metropolitan areas, and differences were generally more marked in women than men. In general, adjusting for SEP did not explain the differences in health risk factors and where it did, effects were modest. For young adults living in Australia, this study suggests that a focus on geographic location as its own social determinant of health beyond SEP is warranted. Furthermore policies and programs may require tailoring for both specific behaviours within non-metropolitan regions and also specific behaviours between males and females living in non-metropolitan areas.
